# Emergency Portasystemic Shunting in Cirrhotics With Bleeding Varices — A Comparison of Portacaval and Mesocaval Shunts

**DOI:** 10.1155/1989/70865

**Published:** 1989

**Authors:** D. I. Soutter, B. Langer, B. R. Taylor, P. Greig

**Affiliations:** 1 Department of Surgery Toronto General Hospital University of Toronto Ontario Toronto Canada; 2 9–237 Eaton North Toronto General Hospital 200 Elizabeth St. Ontario Toronto M5G 2C4 Canada

## Abstract

Despite the best conservative measures available for the control of major variceal hemorrhage, some patients either continue to bleed, or rebleed early, and require emergency surgery. One hundred patients with cirrhosis and uncontrolled bleeding were treated with emergency portasystemic shunts between 1968 and 1983. Fifty eight patients had end-to-side portacaval shunts and 42 had Dacron interposition mesocaval shunts. Both groups were comparable with respect to age, sex and prevalence of alcoholism. There was an increased severity of liver disease as assessed by Child's class in the mesocaval group of patients.

Overall in-hospital mortality was 31% with no significant difference demonstrated between the mesocaval group (28%) and the portacaval group (33%), nor between alcoholic cirrhotics (34%) and non-alcoholic cirrhotics (21%). Mortality rates based on severity of liver disease were: Child's A (1/6) 17%, Child's B (9/48) 19%, and Child’s C (21/46) 46%. There was a statistically significant difference between Child's A & B and Child's C (p < 0.01). Four patients were lost to follow-up. No significant differences were found in 5 year survival by life table analysis comparing portacaval (39%) vs. mesocaval (28%) groups or alcoholic cirrhotics (36%) vs. non-alcoholic cirrhotics (29%). Encephalopathy in survivors was absent in 46%, mild in 28% and severe in 26% of patients. There was no significant difference in encephalopathy rates following portacaval or mesocaval shunting.

Neither operation was clearly superior and choice of operation can be made on the basis of technical and anatomical factors and surgeon experience. Emergency shunting remains a useful option for patients with variceal bleeding refractory to other more conservative therapy, and is associated with acceptable early mortality and long term survival rates.


*HPB Surgery* 1989, Vol. 1, pp. 107–118
Reprints available directly from the publisher
Photocopying permitted by license only

© 1989 Harwood Academic Publishers GmbH
Printed in Great Britain

### EMERGENCY PORTASYSTEMIC SHUNTING IN CIRRHOTICS WITH BLEEDING VARICES — A COMPARISON OF PORTACAVAL AND MESOCAVAL SHUNTS

D.I. SOUTTER, B. LANGER*, B.R. TAYLOR and P GREIG

*Department of Surgery, Toronto General Hospital*
*University of Toronto, Toronto, Ontario, Canada*

*(Received 18 January 1988)*

Despite the best conservative measures available for the control of major variceal hemorrhage, some patients either continue to bleed, or rebleed early, and require emergency surgery. One hundred patients with cirrhosis and uncontrolled bleeding were treated with emergency portasystemic shunts between 1968 and 1983. Fifty eight patients had end-to-side portacaval shunts and 42 had Dacron interposition mesocaval shunts. Both groups were comparable with respect to age, sex and prevalence of alcoholism. There was an increased severity of liver disease as assessed by Child's class in the mesocaval group of patients.

Overall in-hospital mortality was 31% with no significant difference demonstrated between the mesocaval group (28%) and the portacaval group (33%), nor between alcoholic cirrhotics (34%) and non-alcoholic cirrhotics (21%). Mortality rates based on severity of liver disease were: Child's A (1/6) 17%, Child's B (9/48) 19%, and Child's C (21/46) 46%. There was a statistically significant difference between Child's A & B and Child's C ($p < 0.01$). Four patients were lost to follow-up. No significant differences were found in 5 year survival by life table analysis comparing portacaval (39%) vs. mesocaval (28%) groups or alcoholic cirrhotics (36%) vs. non-alcoholic cirrhotics (29%). Encephalopathy in survivors was absent in 46%, mild in 28% and severe in 26% of patients. There was no significant difference in encephalopathy rates following portacaval or mesocaval shunting.

Neither operation was clearly superior and choice of operation can be made on the basis of technical and anatomical factors and surgeon experience. Emergency shunting remains a useful option for patients with variceal bleeding refractory to other more conservative therapy, and is associated with acceptable early mortality and long term survival rates.

KEY WORDS: Bleeding varices, emergency shunts, portocaval shunt, mesocaval shunt

### INTRODUCTION

The treatment of variceal hemorrhage remains a difficult and challenging problem. Most patients stop bleeding either spontaneously or with non-operative management including Pitressin, balloon tamponade and injection sclerotherapy. Some patients however, either continue to bleed, or rebleed after initial control and may require emergency surgical intervention to stop the bleeding. Although portasystemic shunting remains the standard surgical management in this situation[1,2,3], there is less agreement as to which shunt is best. The mesocaval shunt has been preferred by some[4,5,6,7,8] because it is felt to leave a lower encephalopathy rate; and discouraged by others[9,10,11] because of its higher thrombosis rate. This report documents our experience with mesocaval and portacaval shunting for refractory

* Address correspondence to: Dr. B. Langer, 9–237 Eaton North, Toronto General Hospital, 200 Elizabeth St., Toronto, Ontario, M5G 2C4.

variceal bleeding over a 16 year period and examines postoperative mortality, encephalopathy and long term survival.

### METHODS

Between 1968 and 1983, 100 patients with biopsy proven cirrhosis and variceal bleeding underwent emergency portasystemic shunting. These were patients who continued to bleed despite medical management including attempted correction of coagulopathy, IV Pitressin, balloon tamponade and injection sclerotherapy; or had early recurrent uncontrolled bleeding. All patients received at least 6 units of blood prior to operation. Those patients who stopped bleeding were operated on electively (subsequent admission) or urgently (same admission) and some of this group have formed the basis of previous reports[3,12,29]. More recently, injection sclerotherapy has been used as long term primary elective therapy for variceal bleeding with shunt procedures reserved for failures.

Patients were denied emergency shunt surgery if they were comatose or had severe coagulopathy refractory to the administration of coagulation factors. Also excluded were those patients with clinical or histological (> 10% Mallory's hyaline) evidence of alcoholic hepatitis. This group of non-shunted patients received either; no therapy (if moribund), transhepatic coronary vein embolization, establishment of transhepatic portasystemic shunt, or other non-shunt surgical procedures.

In the 100 patients having emergency shunts, pre-operative endoscopy was carried out in 75, liver biopsy in 85 and angiography in 60 patients. The mean age of patients was 54 years with a range of 23 to 76 years. Seventy six patients had alcoholic cirrhosis and 24 had post-necrotic cirrhosis. Hepatic functional reserve was assessed according to the method of Child and Turcotte. Six patients were categorized Child's class A, 48 were considered class B, and 46 were considered class C. All classifications were done during a bleeding episode since only cases of ongoing refractory bleeding form the basis of this report.

Shunt selection was left up to each of three operating surgeons. Each surgeon performed both types of shunts and choice of procedure depended primarily on intra-operative assessment of ease of particular operation. There was a tendency for patients with massive ascites to undergo mesocaval shunting, and this may account in part for the higher number of Child's C patients in the mesocaval group (see below). In addition, 24 patients were entered into a randomized trial of mesocaval versus portacaval shunting for refractory bleeding. These patients have not been separately reported because it was felt that there were insufficient numbers to draw any conclusions.

Fifty eight patients had end-to-side portacaval shunts and 42 patients had H-graft interposition mesocaval shunts using large diameter (18–20 mm) Dacron Weave-knit grafts. Table 1 outlines characteristics of these 2 groups of patients. The only demographic difference between the two groups was a higher proportion of Child's C patients in the mesocaval group.

All patients were followed up in a combined medical surgical shunt clinic. Long term follow-up in less than 10% of cases required correspondence with family or referring physicians. Four patients were lost to follow-up. Encephalopathy was determined at shunt clinic visits and during subsequent hospital re-admissions by history, physical exam and trail testing. Patients with mild abnormalities on trail testing were considered as having mild encephalopathy. Encephalopathy induced

**Table 1** Characteristics of Shunted Patients

| | *Mesocaval* *n=42* | *Portacaval* *n=58* |
|---|---|---|
| | n ( % ) | n ( % ) |
| males | 29 (69%) | 34 (59%) |
| females | 13 (31%) | 24 (41%) |
| Child's class: | | |
| A | 1 ( 2%) | 5 ( 8%) |
| B | 16 (38%) | 32 (55%) |
| C | 25 (60%) | 21 (37%) |
| Alcoholic | 32 (76%) | 44 (76%) |
| Non-alcoholic | 10 (24%) | 14 (24%) |
| age (mean) | 55 years | 54 years |

by recurrent GI bleeding or as part of terminal hepatic failure is not considered in the assessment of functional results. Otherwise we termed encephalopathy as "mild" if easily managed, did not interfere with normal function and did not require hospitalization. Encephalopathy was termed "severe" if it interfered with daily activities or required hospitalization for control.

### RESULTS

### A] *Mortality:*

Overall in-hospital mortality was 31 to 100 patients. This included a few patients who lived longer than 30 days but died in the initial hospitalization. Mortality of alcoholic cirrhotics was 26 of 76 patients (34%) and of non-alcoholic cirrhotics 5 of 24 patients (21%). Although a trend was noted towards improved survival in non-alcoholics it was not statistically significant ($\chi^2 = 0.96$ $p > 0.05$). Mortality based on severity of liver disease as assessed by Child's class is outlined in Table 2. Of six Child's A patients, one died (17%). Nine of 48 Child's B (19%) and 21 of 46 Child's C (46%) died post-operatively. The difference between Child's classes A plus B and Child's class C is statistically significant ($\chi^B = 7.33$, $p < 0.01$). There was no difference in the overall operative mortality of the mesocaval group (12 of 42 patients; 28%) and the portacaval group (19 of 58 patients; 33%). Similarly, there were no differences in the operative mortalities between mesocaval or portacaval groups stratified for Child's classification.

**Table 2** Operative Mortality vs Child's Class and Shunt Type

| | *Mesocaval* | | *Portacaval* | | *Both Shunts* | |
|---|---|---|---|---|---|---|
| | *# pt* *n* | *deaths* *n (%)* | *# pt* *n* | *deaths* *n (%)* | *# pt* *n* | *deaths* *n (%)* |
| Child's A | 1 | 0 ( 0) | 5 | 1 (20) | 6 | 1* (17) |
| B | 16 | 3 (19) | 32 | 6 (19) | 48 | 9* (19) |
| C | 25 | 9 (36) | 21 | 12 (57) | 46 | 21* (46) |
| All PTS: | 42 | 12~(28) | 58 | 19~(33) | 100 | 31 (31) |

* difference between Child's class A & B and class C statistically significant ($\chi^2 = 7.33$, $p < 0.01$)
~ non significant ($\chi^2 = .053$, $p > 0.05$).

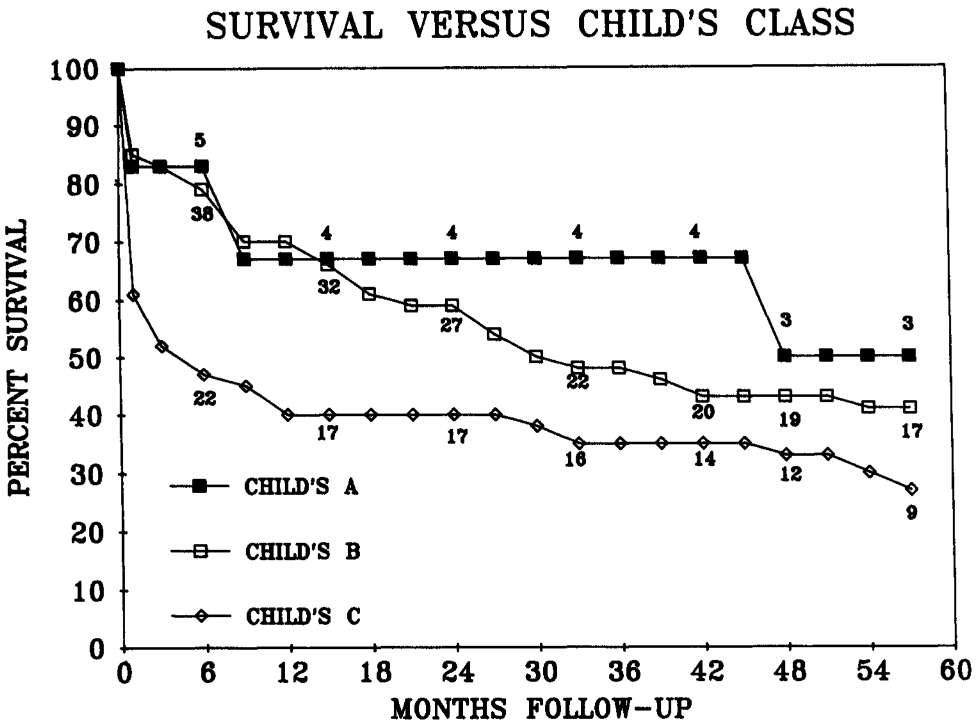

**Figure 1** Kaplan-Meier estimated five year survival versus Child's classification. Child's A — 50%, Child's B — 41%, Child's C — 27%. Difference between Child's B and Child's C is statistically significant by a log rank analysis, DF=1, L=6.96, $P<0.01$. Numbers above graphs represent patients being followed at the beginning of each interval.

B] *Long term survival:*

Analysis of survival is by the actuarial life table method. Different populations are compared by log rank analysis. Survival curves based on Child's class are shown in Figure 1. Estimated 5 year survival figures are: Child's A = 50%, Child's B = 41%, and Child's C = 27%. The difference in survival between Child's B and Child's C is statistically significant (df=1, L=6.96, $P<0.01$). This difference however, is due to the disparity of initial operative mortality as demonstrated by analysis of patients who survived operation and left hospital (Figure 2), where no difference in long term survival is seen. Similarly there was no difference in long term survival found by comparing alcoholic cirrhotics with non-alcoholic cirrhotics (Figure 3), or mesocaval shunted patients with portacaval shunted patients (Figure 4).

C] *Encephalopathy:*

Prior to surgery, a history of chronic encephalopathy was absent in 71 patients. Thirteen patients had historical evidence of mild encephalopathy, and 10 patients had a history of severe encephalopathy. Information is incomplete in the remaining 9 patients. None of the patients had baseline preoperative trail testing prior to their presentation with refractory bleeding.

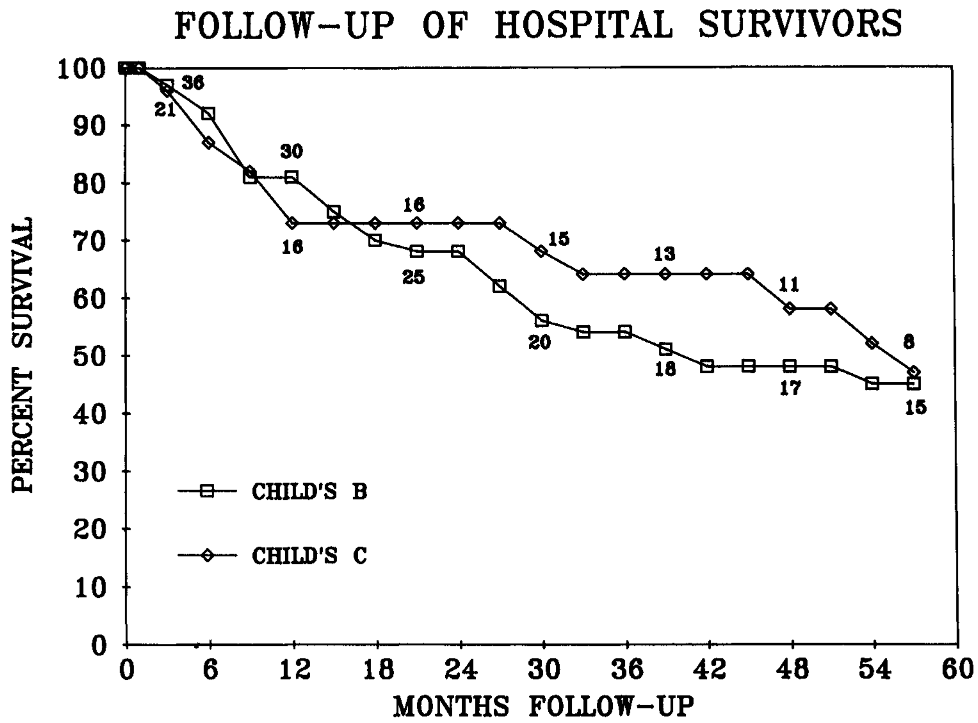

**Figure 2** Long term survival of patients who leave hospital. No significant difference by log rank analysis between Child's B and Child's C patients. Apparent difference in Figure 1 due to operative mortality. Numbers above graphs represent patients being followed at the beginning of each interval.

Sixty five of the 69 hospital survivors have been followed up at the shunt clinic. Thirty patients (46%) have had no encephalopathy. Eighteen patients (28%) have had mild encephalopathy managed as an out-patient with dietary control. Seventeen patients (26%) have had "severe" encephalopathy requiring hospitalization for management, or significantly affecting their normal lifestyle. There has been no difference in the incidence or severity in encephalopathy between the two types of portasystemic shunts as outlined in Table 3. Particularly noted is the incidence of severe encephalopathy of 25% in the mesocaval group as compared with 27% in the portacaval group.

Assessment of change in encephalopathy from pre-operative status is somewhat

**Table 3** Encephalopathy in Hospital Survivors

| | *Mesocaval n=28* | | *Portacaval n=37* | | *Both Shunts n=65* | |
|---|---|---|---|---|---|---|
| | *n* | *(%)* | *n* | *(%)* | *n* | *(%)* |
| ABSENT | 15* | (54) | 15* | (41) | 30 | (46) |
| MILD | 6* | (21) | 12* | (32) | 18 | (28) |
| SEVERE | 7* | (25) | 10* | (27) | 17 | (26) |

* difference not significant ($\chi^2$ = 1.31, df=2, p=.52).

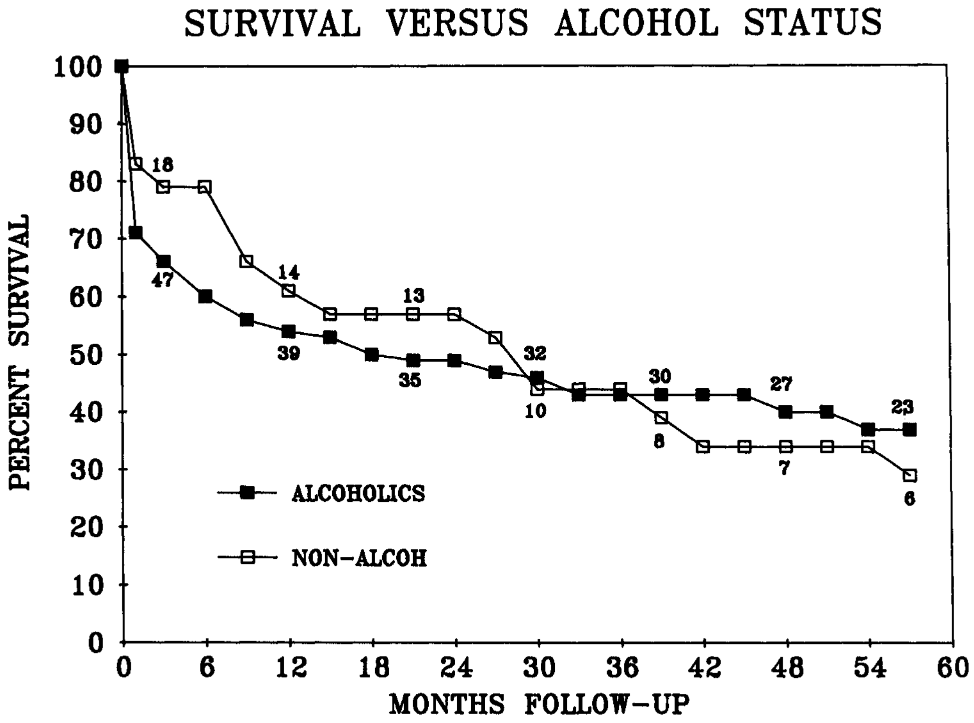

**Figure 3** Kaplan-Meier estimated a five year survival of alcoholic cirrhotics and nonalcoholic cirrhotics. No significant difference by log rank analysis. Numbers above graphs represent patients being followed at the beginning of each interval.

obscured by the fact that results of trail testing are not available pre-operatively for comparison. With this proviso aside, we found that in the 69 patients surviving shunt surgery; 28 (40.6%) were unchanged, 30 (43.4%) worsened, and 6 (8.7%) improved with regards to their pre-bleed mental status. Information is not available in 5 (7.2%) patients.

D] *Rebleeding rates:*

Of the 65 operative survivors followed up on a long term basis, 15 (23%) have been re-admitted with upper G.I. bleeding; however, on investigation, in only 2 (3%) has the bleeding been documented as coming from esophageal varices. Six patients who rebled (including one from varices) were in the 39 patients followed after portacaval anastomosis; and 9 patients who rebled (including the other variceal bleed) were in the 30 patients followed after mesocaval anastomosis.

Post-mortem examinations have been performed in 11 patients in the mesocaval group. Two patients had shunt thrombosis, one of these was the patient who died from recurrent variceal bleeding. Four thrombosed portacaval shunts have been found amongst 15 patients in the portacaval group who have had autopsies.

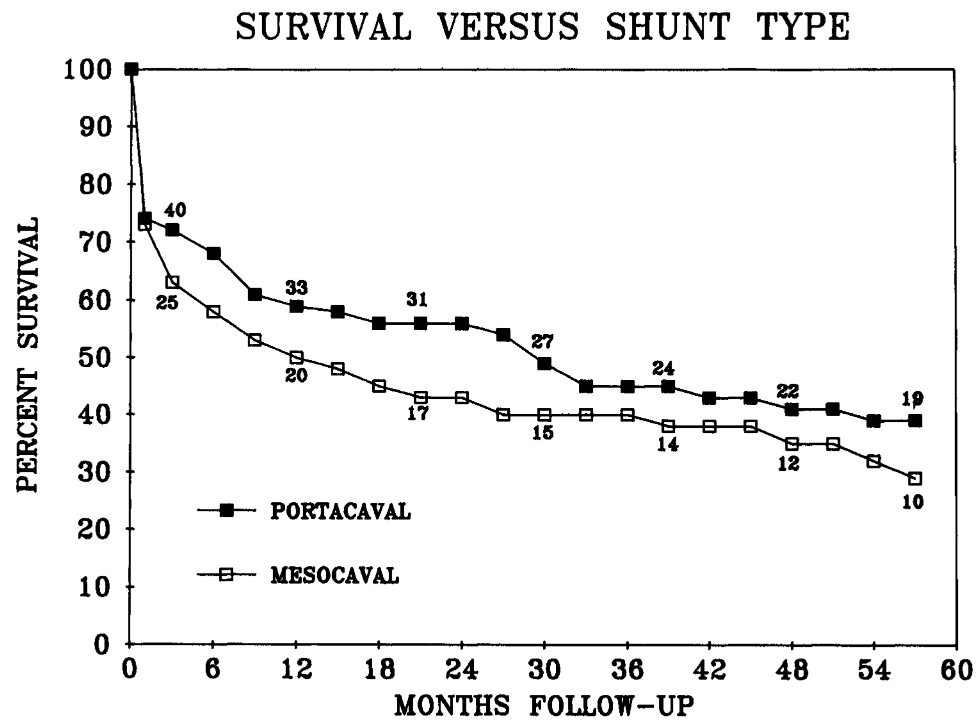

**Figure 4** Kaplan-Meier estimated a five year survival of portacaval and mesocaval shunted patients. No significant difference by log rank analysis. Numbers above graphs represent patients being followed at the beginning of each interval.

### DISCUSSION

Is there still a role for emergency portasystemic shunting in cirrhotics with variceal bleeding? There is a lack of stastical evidence to support improved long term survival after portasystemic shunting[13,14,15,16], and most reports indicate significant mortality rates (20–82%) in patients undergoing emergency shunt operations[11,17,18,19]. These factors along with recent data demonstrating improved control of variceal bleeding using esophageal sclerotherapy[20,21], has led to a reluctance to use emergency shunt surgery. There is however, still a significant incidence of failure of medical management (including sclerotherapy), with persistant hemorrhage contributing to patient death as reported in sclerotherapy arms of recent controlled trials. Cello[22] described 13 of 28 patients (47%) who died due to bleeding causes despite sclerotherapy. MacDougall[20] attributed 4 deaths in 51 sclerosed patients (8%) to refractory hemorrhage. In Rikker's trial[23] of sclerotherapy vs distal splenorenal shunt, 6 of 30 patients in the sclerotherapy arm died of bleeding. It is these patients who fail medical management that should be considered for emergency surgery.

Other surgical approaches to the emergency control of bleeding are still unproven. Esophageal transection procedures[24,25] have operative mortality rates as high as shunts and as yet undetermined long term rebleeding rates. The extensive devascularization described by Sugiura[26] has a low operative mortality and encephalopathy rate based on a non-alcoholic Japanese population, but has not been duplicated in the North American setting. We have considered as unsuitable for operation most patients with frank coma, uncorrectable coagulopathy, hepatorenal failure or clinical or histological severe alcoholic hepatitis. These patients are treated with transhepatic coronary vein embolization, transhepatic radiological shunt, esophageal transection or devascularization.

Despite this selection, our mortality in Child's C patients remains 46%. The salvage in these 46 poor risk patients however, was satisfactory with a projected 5 year survival rate of 27%; two Child's C patients are still alive at 7 and 10 years.

There was no demonstrable difference in operative mortality of alcoholic cirrhotics (34%) and non-alcoholic cirrhotics (21%). Similarly, we found no difference in long term survival between non-alcoholic and alcoholic cirrhotics after emergency shunting. Although some have found improved long term survival in non-alcoholics after effective distal splenorenal shunt[27,28], this does not appear to hold true after total shunts[29].

Encephalopathy in survivors was present in 54% of patients, and was similar in portacaval and mesocaval shunt patients. We have previously shown that the mesocaval shunt functions as a total shunt hemodynamically[30] and one would not expect any difference in post-shunt encephalopathy rates. This rate is similar to that reported by others after total shunting[10]. In 26% of patients encephalopathy interfered with activities of daily living or required hospitalization, but 74% of patients followed had either no encephalopathy (46%) or little disability (28%). We have not been able to predict the occurrence of "severe" encephalopathy by any pre-op parameter in any of this group of emergency shunted patients, or other groups reported from this institution[3,12].

We feel therefore, that there remains an important role for emergency portasystemic shunting in patients who are truly refractory to non-operative therapies to stop their bleeding, and that persistant use of non-surgical therapy in such patients is not warranted.

There remains controversy as to which type of shunt is best suited for emergency portasystemic decompression. The interposition mesocaval shunt was enthusiastically adopted in the 1970's, because of reports suggesting that it had a lower encephalopathy rate[5,7,8]. A randomized trial in the elective setting did not bear out this claim[9]. There has been only one randomized trial of emergency interposition mesocaval shunting which "favoured portacaval shunting" but the numbers were small and the data influenced by an unexplained high (72%) operative mortality rate in the mesocaval shunt patients, most of whom were Child's class A or B[11]. Most other authors report far less operative mortality with emergency mesocaval shunting[4,5,6,10], and are in close agreement to the rate of 28% in this series. Although we found no difference between this operative mortality and the rate of 33% in the portacaval group, the groups had unequal numbers of Child's class B and C patients. Survival should have been prejudiced against the mesocaval group which had more class C and fewer class B patients. In fact, the mortality rate of Child's C patients was lower in the mesocaval group (36%) than in the portacaval group (57%); but the differences are not significant.

The main disadvantage of the mesocaval shunt is the reported incidence of late shunt thrombosis and variceal rebleeding[7,10,31]. Although we have studied most patients with early post-operative angiography, we do not have long term radiological follow-up except where clinically indicated. In our mesocaval group however, only 1 of 30 patients who left hospital later rebled from varices; and of 11 patients who had an autopsy, 2 had shunt thrombosis. It does not seem that repeated variceal bleeding after mesocaval shunting has been a major clinical problem and this fact is also borne out by a similar 5 year survival with the portacaval shunted patients.

In summary, we found no significant difference in the operative mortality, long term survival or incidence of encephalopathy between these two types of shunts in 100 patients operated on as an emergency. There are certain situations when a mesocaval shunt is preferable to an end-to-side portacaval shunt. These include patients with intractable ascites, reversal of portal flow, and where technical considerations make portacaval shunting extremely difficult. Where these situations do no exist, our current recommendation would be to do the shunt with which the surgeon has the most experience or which is technically easier in the individual patient.

### *References*

1. Malt R.A., Portasystemic venous shunts. *NEJM* 1976; **295:** 24–29, 80–86.
2. Rikkers, L.F., Operations for management of esophageal variceal hemorrhage (Medical Progress). *West J. Med.* 1982; **136:** 107–21.
3. Langer B., Patel S.C., Stone R.M., *et al:* Selection of operation in patients with bleeding esophageal varices. *CMA Journal* 1978; **118:** 369–72.
4. Dowling, J.B., Ten years' experience with mesocaval grafts. *Surg. Gynaecol. Obstet.* 1979; **149:** 518–22.
5. Thompson B.W., Casali R.E., Read R.C., Campbell G.S., Results of interposition "H" grafts for portal hypertension. *Ann. Surg.* 1978; **187:** 515–22.
6. Reichle F.A., Fahmy W.F., Golsorkhi M., Prospective comparative clinical trial with distal splenorenal and mesocaval shunts. *Am. J. Surg.* 1979; **137:** 13–21.
7. Cameron J.L., Zudiema G.D., Smith G.W., *et al:* Mesocaval shunts for the control of bleeding esophageal varices. *Surgery* 1979; **85:** 257–62.
8. Drapanas T., LoCicero J., III, Dowling J.B., Hemodynamics of the interposition mesocaval shunt. *Ann. Surg.* 1975; **181:** 523–33.
9. Stipa S., Ziparo V., Anza M., Fabrini G., Lupino R., A randomized controlled trial of mesentericocaval shunt with autologous jugular vein. *Surg. Gynaecol. Obstet.* 1981; **153:** 353–6.
10. Smith R.B., Warren W.D., Salem A.A., *et al:* Dacron interposition shunts for portal hypertension. An anlysis of morbidity correlates. *Ann. Surg.* 1980; **192:** 9–17.
11. Malt R.A., Abbott W.M., Warshaw A.L., *et al:* Randomized trial of emergency mesocaval and portacaval shunts for bleeding esophageal varices. *Am. J. Surg.* 1978; **135:** 584–88.
12. Langer B., Taylor B.R., Mackenzie D.R., *et al:* Further report of a prospective randomized trial comparing distal splenorenal shunt with end-to-side portacaval shunt *Gastroenterology* 1958; **88:** 424–9.
13. Jackson F.C., Perrin E.B., Felix W.R., Smith A.G., A clinical investigation of the portacaval shunt: V. Survival analysis of the therapeutic option. *Ann. Surg.* 1971; **174:** 672–701.
14. Mikkelson W.P., Therapeutic portacaval shunt Preliminary data on controlled trial and morbid effects of acute hyaline necrosis. *Arch. Surg.* 1974; **108:** 302–5.
15. Resnick R.H., Iber F.L., Ishihara A.M., *et al:* A controlled study of the therapeutic portacaval shunt. *Gastroenterology* 1974; **67:** 843–57.
16. Rueff B., Prandi D., Degos F., *et al:* A controlled study of therapeutic portacaval shunt in alcoholic cirrhotics. *Lancet* 1976; **1:** 655–59.
17. Rueff B., Benhamou J.P., Management of gastrointestinal bleeding in cirrhotic patients. *Clinics in Gastroenterology* 1975: **4(2):** 425–38.
18. Orloff M.J., Bell R.H., Hyde P.V., *et al:* Long term results of emergency portacaval shunt for bleeding esophageal varices in unselected patients with alcoholic cirrhosis. *Ann. Surg.* 1980; **192:** 325–340.
19. Cello J.P., Deveney K.E., Trunkey D.D., *et al:* Factors influencing survival after therapeutic shunts. Results of a discriminant function and linear logistic regression analysis. *Am. J. Surg.* 1981; **141:** 257–65.

20. Macdougall B.R.D., Westaby D., Theodossi A., *et al:* Increased long term survival in variceal hemorrhage using injection sclerotherapy. Results of a controlled trial. *Lancet* 1982; **1:** 124–7.
21. Terblanche J., Bornman P.C., Kahn D., *et al:* Failure of repeated injection sclerotherapy to improve long term survival after oesophagael variceal bleeding. *Lancet* 1983; **2:** 1328–32.
22. Cello J.P., Grendell J.H., Crass R.A., *et al:* Endoscopic sclerotherapy versus portacaval shunt in patients with severe cirrhosis and variceal hemorrhage. N.E.J.M. 1984; **311:** 1589–94.
23. Rikkers L.F., Burnett D.A., Volentine G.D., Shunt surgery versus sclerotherapy for long term treatment of variceal bleeding. Early results of a randomized trial. *Ann. Surg.* 1987; **206:** 261–271.
24. Wexler M.J., Treatment of bleeding esophageal varices by transabdominal esophageal transection with the EEA staplin instrument. *Surgery* 1980; **88:** 406–16.
25. Cello J.P. Crass R., Trunkey D.D., Endoscopic sclerotherapy versus transection in Child's class C patients with variceal hemorrhage. Comparison with results of portacaval shunt: *Surgery* 1982; **91:** 333–38.
26. Sugiura M., Futagawa S., Further evaluaton of the Sugiura procedure in the treatment of esophageal varices. *Arch. Surg.* 1977; **112:** 1317–21.
27. Zeppa R., Hensley G.T., Levi J.U. *et al:* The comparative survival of alcoholics versus non-alcoholics after distal splenorenal shunt. *Ann. Surg.* 1978; **187:** 510–14.
28. Warren D.W., Millikan W.J. Henderson M.J. *et al:* Ten years portal hypertensive surgery at Emory. *Ann. Surg.* 1982; **195:** 530–42.
29. Soterakis F., Resnick R.H., Iber F.L., Effect of alcohol abstinence on survival in cirrhotic portal hypertension. *Lancet* 1973; **2:** 65–7.
30. Reznick R.K., Langer B., Taylor B.R., *et al:* Results and hemodynamic changes after interposition mesocaval shunt. *Surgery* 1984; **95:** 275–79.
31. Dennis M.A., Monson R.C., O'Leary J.P., Interposition mesocaval shunt: a less than ideal procedure. *American Surgeon* 1978; **44:** 734–38.

*Accepted by S. Bengmark on 13 June 1988.*

### INVITED COMMENTARY

In this issue of HPB Surgery, Soutter and colleagues[1] from the University of Toronto present their experience with 100 emergency portal-systemic shunts accumulated over an interval of 15 years. These surgeons adhered to a policy of selective emergency surgery performed only after intravenous vasopressin infusion, balloon tamponade, and in more recent years, endoscopic sclerotherapy had failed to control acute variceal hemorrhage. End-to-side portacaval and interposition mesocaval shunts were utilized with shunt selection based mainly on the presence or absence of advanced ascites. Despite more Child's C patients undergoing the interposition shunt, overall results were approximately equivalent after both procedures with respect to operative mortality, long-term survival, postoperative encephalopathy, and recurrent variceal hemorrhage. As would be expected with totally diverting, nonselective shunts, encephalopathy complicated the postoperative courses of 54% of patients and was severe in 26% of patients. Considering prior publications[2] reporting a high incidence of late shunt thrombosis after the interposition mesocaval shunt, the frequency of recurrent hemorrhage was surprisingly low with only one case documented in each shunt group. Compatible with numerous prior reports, operative mortality rates were related to Child's class rather than to the procedure selected. Even though many of the patients were desperately ill, long-term salvage was impressive in patients with both alcoholic and nonalcoholic cirrhosis. This excellent series from Toronto rekindles two long standing controversies regarding emergency surgery for patients with bleeding varices: 1) When is the optimal time for surgical intervention? and 2) What is the preferred operative procedure in the emergency setting?

In most centers, emergency surgery has played less of a role in management of acute variceal hemorrhage as more effective nonoperative interventions, such as endoscopic sclerotherapy, have become available. The majority of portal hypertension surgeons would agree with a policy of selective emergency surgery similar to the one presented by Soutter and associates. Whenever possible, acute bleeding should be controlled with nonoperative means, allowing time for optimization of hepatic function and nutritional status prior to elective surgery. An interval of medical management may be especially important in patients with alcoholic hepatitis, who may be at particularly high risk for postoperative morbidity or mortality.[3] One study has shown that Child's class, and therefore, operative risk, can be significantly improved when a period of conservative treatment precedes surgery.[4] With the availability of endoscopic sclerotherapy in most institutions, acute episodes of variceal hemorrhage can generally be controlled without surgery in more than 80% of instances.

Orloff is one of the few remaining advocates of routine emergency surgery for all patients who bleed from varices. He believes that the maximum number of patients can be salvaged by operating within eight hours of admission to the hospital before adverse consequences of hemorrhage such as further hepatic functional deterioration ensue. He has reported an overall operative mortality rate of 42% (17% in recent years) in 180 consecutive emergency portacaval shunts.[5] Orloff's group has conducted the only randomized, controlled trial of conservative management followed by elective shunt surgery versus emergency portacaval shunt for acutely bleeding patients.[6] The results of their trial, which have only been published in abstract form, reveal superior bleeding control (100% vs 45%) and lower early mortality (19% vs 55%) in the emergency portacaval shunt group. However, sclerotherapy was not used in the conservative treatment arm of the study and the two nonoperative treatments used, vasopressin infusion and balloon tamponade, were considerably less effective than in most other series. Therefore, despite the results of this investigation, selective emergency surgery, reserved for those few patients who fail all nonoperative attempts at control of acute variceal bleeding, rather than routine emergency surgery is preferred by the majority of physicians and surgeons who treat this challenging problem.

When emergency surgery is required, it remains controversial as to which procedure should be done. I believe that an important guiding principle is that an individual surgeon should select a familiar operation which can be completed expeditiously. The emergency setting is not the time to learn or experiment with new procedures. Because of its relative simplicity and the brief operating time required, esophageal transection with the end-to-end anastomosis (EEA) stapler is the emergency operation of choice for many surgeons. However, there is little evidence that this procedure is followed by a lower operative mortality rate than shunt operations and significant disadvantages of esophageal transection include a high frequency of recurrent hemorrhage, complete ineffectiveness in patients with bleeding gastric varices or portal hypertensive gastropathy, and a potentially tenuous stapled anastomosis in patients who have received recent, intensive sclerotherapy.[7]

The most frequently used emergency shunts are the end-to-side portacaval shunt and the interposition mesocaval shunt, the procedures selected by Soutter and colleagues. Although they found these operations to be approximately equivalent, others have reported more frequent early and late recurrent hemorrhage after the interposition mesocaval shunt.[2,8] Both of these shunts totally divert portal flow and are, therefore, frequently complicated by postoperative encephalopathy. Since the interposition shunt decompresses hepatic sinusoids as well as the splanchnic venous circulation, it is more effective for relief of postoperative ascites than the end-to-side portacaval shunt. Other potential advantages of the interposition procedure are avoidance of dissection in the porta hepatis, making subsequent liver transplantation more feasible, and easy reversibility if intractable encephalopathy should develop.

When surgery is urgent rather than emergent (e.g. bleeding temporarily controlled by balloon tamponade), I believe that a distal splenorenal shunt, which has the potential of preserving hepatic portal perfusion in addition to decompressing varices, is a reasonable alternative. If the surgeon is experienced in performing this procedure, the operative mortality rate appears to be similar to other emergency operations.[9] Visceral angiography should precede surgery in all patients undergoing the distal splenorenal shunt, and this operation should not be selected for patients with ascites intractable to medical management or with a splenic vein less than 8 mm in diameter. Although some patients with a patent selective shunt may develop recurrent hemorrhage secondary to renal vein hypertension, bleeding is usually not life-threatening and is limited to the early postoperative interval.

### *References*

1. Soutter D.I., Langer B., Taylor B.R., *et al:* Emergency portasystemic shunting in cirrhotics with bleeding varices--a comparison of portacaval and mesocaval shunts. *HPB Surgery* (in press.)
2. Smith R.B., Warren W.D., Salam A.A., *et al:* Dacron interposition shunts for portal hypertension. *Ann Surg* **192:9**, 1980.
3. Eckhauser F.E., Appelman H.D., O'Leary T.J., *et al:* Hepatic pathology as a determinant of prognosis after portal decompression. *Am. J. Surg.* **139:**105, 1980.
4. Holman JM, Rikkers LF: Success of medical and surgical management of acute variceal hemorrhage. *Am. J. Surg.* 140:816, 1980.
5. Orloff M.J., Bell R.H. Jr, Hyde P.V., *et al:* Long-term results of emergency portacaval shunt for bleeding esophageal varices in unselected patients with alcoholic cirrhosis. *Ann Surg* **192:**325, 1980.
6. Orloff M.J., Bell R.H. Jr, Greenburg AG: Prospective randomized trial of emergency portacaval shunt and medical therapy in unselected cirrhotic patients with bleeding varices. *Gastroenterology* **90:**1754, 1986.
7. Cello J.P., Crass R., Trunkey D.D.: Endoscopic sclerotherapy versus esophageal transection in Child's class C patients with variceal hemorrhage. Comparison with results of portacaval shunt: preliminary report. *Surgery* **91:**333, 1982.
8. Malt R.A., Abbott W.M., Warshaw A.L., *et al:* Randomized trial of emergency mesocaval and portacaval shunts for bleeding esophageal varices. *Am. J. Surg.* **135:** 584, 1978.
9. Potts J.R. III, Henderson JM, Millikan WJ Jr, *et al:* Emergency distal splenorenal shunts for variceal hemorrhage refractory to nonoperative control. *Am. J. Surg.* **148:** 813, 1984.

Layton F. Rikkers
Department of Surgery
University of Nebraska Medical Center
Omaha, Nebraska, USA

